# Microstructure, Microhardness, Corrosion Resistance and Chemical Composition of Mo, B and Mo-B Coatings Produced Using Laser Processing

**DOI:** 10.3390/ma13153249

**Published:** 2020-07-22

**Authors:** Aneta Bartkowska, Dariusz Bartkowski, Mikołaj Popławski, Adam Piasecki, Damian Przestacki, Andrzej Miklaszewski

**Affiliations:** 1 Institute of Materials Science and Engineering, Faculty of Materials Engineering and Technical Physics, Poznan University of Technology, ul. Jana Pawła II 24, 61-138 Poznan, Poland; mikolaj.poplawski@put.poznan.pl (M.P.); adam.piasecki@put.poznan.pl (A.P.); andrzej.miklaszewski@put.poznan.pl (A.M.); 2 Institute of Materials Technology, Faculty of Mechanical Engineering, Poznan University of Technology, ul. Piotrowo 3, 61-138 Poznan, Poland; dariusz.bartkowski@put.poznan.pl; 3 Institute of Mechanical Technology, Faculty of Mechanical Engineering, Poznan University of Technology, ul. Piotrowo 3, 61-138 Poznan, Poland; damian.przestacki@put.poznan.pl

**Keywords:** molybdenum, boron, laser alloying, microhardness, corrosion resistance, XRD, EDS

## Abstract

The paper presents study results of laser alloying of CT90 tool steel with an applied pre-coat of boron, molybdenum or a mixture of these elements. Pre-coats were applied on steel substrates in the form of a paste. The aim of the study was to investigate the microstructure, chemical and phase composition, microhardness and corrosion resistance of these newly-formed coatings. The laser alloying process was carried out using a diode laser with a nominal power of 3 kW. In this study a laser beam power of 900 W and a scanning speed of 48 mm/s were used. As a result of the laser beam action, the presence of three areas was observed in cross-sections of specimens: a remelted zone, a heat affected zone and the substrate. The properties of coatings enriched with both molybdenum and boron were better than those of the steel substrate, but only the use of a Mo-B mixture resulted in a significant improvement in microhardness and corrosion resistance.

## 1. Introduction

The problem of surface modification is an important aspect of material engineering. In order to optimize the production process and obtain required properties of the surface layer, numerous studies on various methods and techniques of surface treatment have been conducted. Diffusion processes are most frequently used methods of surface modification in industry. Interesting properties of layers are obtained using thermo-chemical treatment involving diffusion saturation of the coatings with boron [1,2]. The boronizing process executed on a steel substrate is characterized by obtaining layers with high hardness (up to 1800 HV) and good wear resistance. A typical diffusion boronized layer is composed of two phases: FeB and Fe_2_B. The FeB phase has higher brittleness [3], which may be manifested by spalling and flaking from the substrate, therefore it is modified by various methods [4,5,6,7].

Laser technologies create new possibilities in the modification of boron-containing layers. The advantages of laser processing include high power density as well as the ability to regulate it accurately, precise heat delivery to selected areas, and from ecological point of view no need to use an additional cooling medium. Laser alloying leads to the formation of a coating that differs in microstructure, chemical and phase composition as well as mechanical and physical properties from the base material and the alloying material. To modify the surface layer using laser heat treatment, not only boron but also elements such as nickel, chromium, silicon, copper and molybdenum are used. There are numerous papers on modifying the surface with laser beam. Laser beam can remelt the surface layer formed during the diffusion or galvanic processes or produced in the form of pastes containing various elements.

Production of modern surface layers aims to improve properties of substrate material. In modern and high-performance processing technologies different laser types with various beam powers are used. Laser technology enables among others: hardening [8,9], remelting [5,6,7] or alloying with different elements and phases [10,11,12,13,14,15,16,17,18,19].

The topics of surface layer modifications using laser (laser remelting and laser alloying) using boron are frequently discussed. The main goal of scientists is to develop technology that reduces brittleness of boronized layers, which is their main disadvantage [3,4,5,6,7]. The results of laser surface modification of borided layers produced on the 41Cr4 steel were presented by Pertek and Kulka [5]. After laser modification the following changes in microstructure were observed. Firstly, creation of remelted zone which consisted of eutectic mixture of borides and martensite. Secondly, creation of a heat affected zone containing martensite. It should be noted that the modification did not take place throughout the specimen and substrate with unchanged microstructure can be distinguished. The layer obtained in this way has a lower microhardness than the diffusion boronized layer before modification. Reduction of hardness was beneficial, because the hardness gradient between the surface and the substrate was lower. This was due to the fact that an intermediate zone (heat affected zone) occurred. After laser remelting of boronizing layer the α-Fe, FeB, Fe_2_B and Fe_3_B phases were identified [5,6,7]. The zones resulting from laser modification process consisted of a solid solution, a boride eutectic, and primary borides in various combinations, which were dependent on the laser parameters used. As the microstructure changes, the mechanical properties also change. For example, it was found that wear resistance increases with growing amount of eutectic or borides.

Not only is laser melting of the boronized layer possible, but also laser alloying of the surface with boron. In [15], the boronized layer was produced on EN25 steel by laser boriding. As a result of this process, a eutectic microstructure in the melted zone consisting of iron borides and martensite with a hardness of 1170–1315 HV0.5 was obtained. The authors confirmed the formation of FeB, Fe_2_B and Fe_3_B iron borides, and the martensite phase in the form of Feα was identified. Corrosion tests showed that laser boronized specimens had higher corrosion resistance than EN25 steel. The authors found that the increased corrosion resistance is affected by the amount of boron eutectics, which depends on the laser processing parameters used. Morimoto et al. also obtained favorable properties. Boron is shown to improve frictional wear resistance while maintaining high hardness [10]. However, in the microstructure, it was found some porosity and several cracks parallel to the beam axis movement. In another work, the microstructure of the Ti6Al4V titanium alloy with boron additives produced by the SLM method was studied [17]. It was found that 2–5 wt.% of boron content allows to obtain high microhardness. In another work, laser surfacing of AISI 1020 steel with an ultra-fine eutectic coating containing boron (Fe-Nb-B) was proposed [18]. As a result of this process researchers obtained a coating composed of a mixture of α-Fe dendrites and borides with a microhardness not exceeding 760 HV0.5. The results presented in the cited paper showed that controlling of laser parameters can contribute to production of coatings characterized by relatively high hardness. Modifying layer containing boron is more and more often carried out with the participation of an additional input of chemical elements which are intended to overcome any negative properties of boron layers such as brittleness.

Laser alloying processes are not as widespread as laser hardening processes [9]. That is why research and development are still focused on this area. During laser alloying, the phenomena occurring are slightly more complicated due to the input of additional elements or compounds to the substrate material.

It is interesting to modify the surface layers e.g., with molybdenum. For example, a plasma alloying method was developed, which involves simultaneous boriding and molybdenizing [4]. As a result of this process, a layer with a thickness of approx. 50 μm and hardness of 13.09 GPa was obtained. This layer consisted of an internal Mo-B diffusion layer and an external Mo-Fe-B composite coating. Hybrid treatment resulted in very good adhesion due to metallurgical bonding. In paper [11], a Fe-based coating containing 13.0% Cr, 1.6% B, 1.2% Si and 0.15% C was produced on the surface of Q235 steel using a 6 kW fiber laser. The effect of molybdenum added in quantities of 0, 1, 2, 3 and 4 wt.% was analyzed. The authors stated that the increase in Mo content increases the wear resistance of the surfacing layer by up to 2.4 times compared to the surfacing layer without molybdenum. However, no significant increase in hardness with the increase of Mo in the surfacing coating was observed.

In [13] molybdenum and zirconium oxide pastes were applied on NiTi alloy, which were then modified using laser beam. The obtained surface coatings were characterized by lack of cracks and porosity. The microhardness of remelted zone was 660 HV for the coating of Ni-Ti-Mo, and 720 HV for the coating of Ni-Ti-ZrO_2_. It should be noted that the authors managed to get improved wear resistance of NiTi alloy used.

In [20] Cr-CrB_2_ and Mo-MoB coatings were produced by plasma spraying and laser alloying. The authors found that Mo-based coating provides low friction coefficient, but unfortunately has a low microhardness. Plasma sprayed coatings are porous and have low adhesion. Therefore, in this study [20], additional laser modification was used. Cr-CrB_2_ and Mo-MoB coatings were applied on AISI 4130 steel by plasma spraying, and next remelted by laser beam. The resulting coatings had microhardness in the range of 1100 HV.

In another article [14], the authors compared plasma spraying and laser alloyed Mo and Mo + Ni, and Cr coatings. It was found that plasma sprayed coatings were porous and had low adhesion to the steel substrate, therefore the coatings were modified by using laser beam. Laser heat treatment improved the overall band strength and hence tribological performance.

Analyzing the available papers, it can be concluded that there are very few publications about manufacturing Mo-B coatings on steel by alloying with a laser beam. A significant part of these papers concerns surface modification with boron or molybdenum alone, therefore it seems justified to raise this issue.

In this study a laser alloying of surface layer of CT90 steel was investigated. The effects of various coat materials (Mo, B and mixtures of B and Mo) with constant laser parameters were analyzed. The microstructure, microhardness, corrosion resistance as well as chemical and phase composition of new and previously untested Mo, B and Mo-B coatings were investigated and compared.

## 2. Materials and Methods

Specimens in the form of plates (20 mm × 12 mm × 4 mm) made of the CT90 steel were used. The chemical composition of the steel used was investigated using Solaris CCD PLUS optical emission spectroscope and presented in Table 1.

It is a cold work tool steel containing the least amounts of alloying additions among tool steels. Microstructure of this steel in the normalized state is perlite grains surrounded by cementite network.

Before laser alloying of surface, specimens were hardened in water from 780 ℃ and tempered at 560 ℃ for 1 h. Subsequently, the specimens were cleaned of any oxides, then degreased using acetone, and finally a pre-coat was applied.

The pre-coats were in the form of a paste containing modifying chemical elements (Mo and B). To modify, boron, molybdenum as well as mixture which contained of 50% molybdenum and 50% boron were used. The paste components were weighed on an AS 60/220.R2 PLUS analytical balance (RADWAG, Radom, Poland) and mixed in the proportions listed in Table 2.

**Figure 1:**
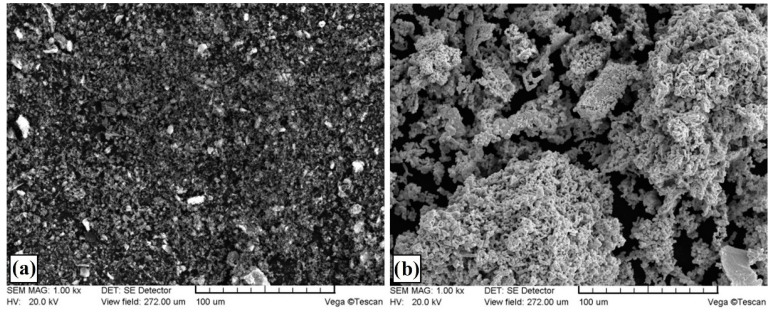
Powder morphologies of boron (**a**) and molybdenum (**b**).

Pre-coats with selected chemical elements were prepared using a mixture of a saturating element selected, sodium water glass and distilled water. Such a mixture in the right proportions ensures a good consistency for applying the pre-coat to the steel substrate and its good adhesion. Both boron and molybdenum pre-coats were 100 µm thick and contained amorphous boron or metallic molybdenum powder as well as and abovementioned water glass and distilled water. Initial studies have shown that thicker pre-coats cause numerous cracks in the produced coating after laser modification. Pre-coats consisting of molybdenum and boron mixture were characterized by the same thickness. The morphology of boron and molybdenum powders are shown in Figure 1.

The thicknesses of pre-coats were determined based on the average of 10 measurements using ultrasonic thickness gauge PosiTector 6000 (DeFelsko, Ogdensburg, NY, USA). Thickness measurements were carried out with accuracy in the range of +/− 5 μm.

Laser alloying of surface with applied pre-coats was carried out using Trumpf TruDiode 3006 diode laser with nominal power of 3.0 kW (TRUMPF, Ditzingen, Germany). Parameters used in the experiment were: laser beam power P = 900 W, laser beam diameter with a multiple mode (TEM_00_) of circular cross-section d = 1 mm, and scanning laser beam velocity v = 48 mm/s. Based on these data, laser beam radiation density was designated which was equal to q = 114.65 kW/cm^2^.

**Figure 2:**
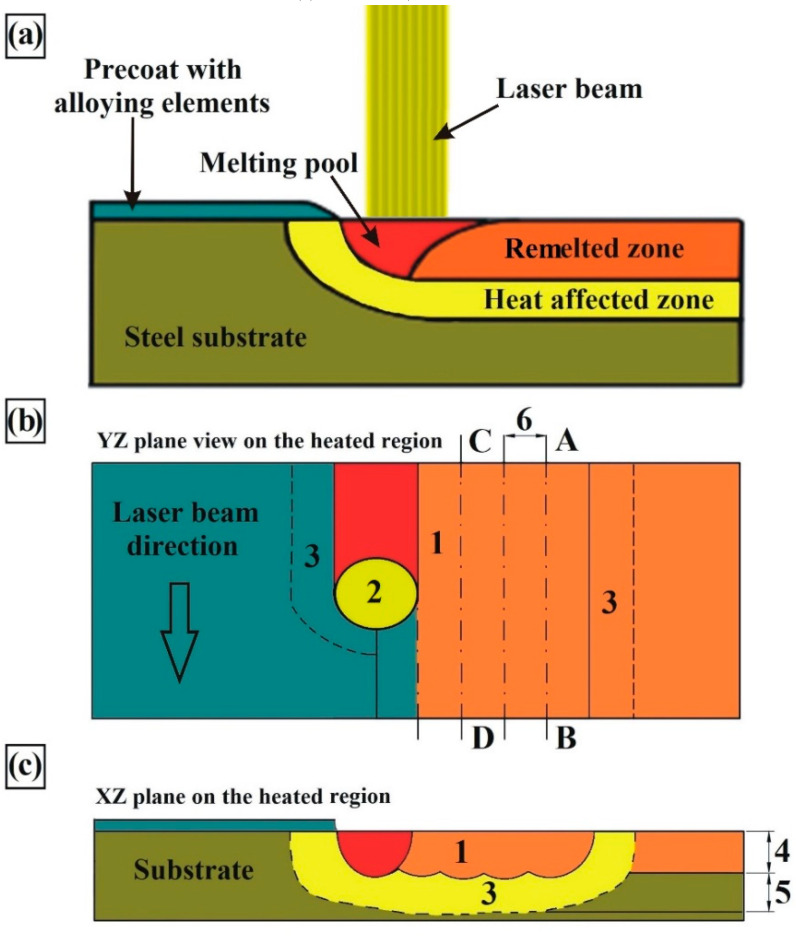
Scheme of laser alloying of steel substrate (**a**), YZ plane on the heat region (**b**), XZ plane on the heat region (**c**), 1—remelted zone, 2—laser beam, 3—heat affected zone, 4—remelted zone depth, 5—heat affected zone depth, 6—laser tracks overlapping.

The laser alloying processes were conducted on the research stand which was composed of diode laser interworking with KR16-2 robotic arm (KUKA, Augsburg, Germany). The possibility of robot operation in several axes allows to perform laser heat treatment of products of various and complex shapes. The procedure used during laser alloying of surface with pre-coat is presented in Figure 2a. Laser tracks were arranged as multiple tracks with distance f = 0.5 mm, where f was the distance between axes of adjacent tracks (Figure 2b). For the produced laser tracks, their overlap was calculated using the equation in which laser beam diameter and distance between the axes of adjacent tracks were taken into account. For the parameters used the tracks overlapping was equal to 50%. Laser tracks were prepared by interaction of the laser beam with the surface of steel used. During the laser treatment process the laser beam was moved from point A to B, then was turned off. Afterwards laser head returned to point A. In the next step the laser beam was transferred by a distance of 0.5 mm and further laser tracks were made from point C to D. This was repeated until the entire surface of the specimen was modified. Distance from the optic lens to the surface of steel was equal to 270 mm. Due to the safety of laser fiber and possibility of a reflection, the laser beam is inclined in XY plane at an angle of 3° [6]. Figure 2b,c shows view in the plane of YZ and XZ respectively. In order to determine the laser beam exposure time on the material as well as laser beam fluence the formulas based on paper [6,21] were used. Exposure time of laser beam on material in expressed in seconds was appointed from formula *E_t_ = d/v*, where *d* was laser beam diameter [mm] and *v* was scanning speed of laser beam [mm/s]. The laser beam fluence was calculated using formula *F = (P·E_t_)/(π·r^2^)*, where *F*—laser beam fluence [J/mm^2^], *P*—laser beam power [W], *r*—radius of the laser beam [mm] *E_t_*—exposure time of laser beam on material [s].

Based on the formulas, it was found that the laser beam exposure time on the material was equal to 0.02 s. At this time, the maximum energy density obtained (fluence) was 22.93 J/mm^2^.

Microstructure observations were carried out using TESCAN VEGA 5135 scanning electron microscope (TESCAN, Brno, Czech Republic). The chemical composition analysis of the obtained coatings was performed using MIRA3 scanning electron microscope equipped (TESCAN) with an EDS-UltimMax energy dispersive spectrometer (Oxford Instruments, High Wycombe, UK) and Aztec Energy Live Standard software. Before observations specimens were cut, ground with abrasive papers of different granularities and then polished with diamond suspension. Finally, specimens were etched in 2% HNO_3_ solution.

The phase composition of the specimens was analyzed using an EMPYREAN PANalytical X-ray diffractometer (PANalytical, Malvern, UK) operating in the angle range of 20–90°. Specimens were investigated using Cu Kα radiation. The LFF focal point (0.4 × 12 mm), Kβ Ni radiation filter as well as 45 kV and 40 mA were applied. The tests were carried out at 25 ℃. Goniometer (Theta/Theta) and minimum step size 2 Theta equal to 0.0001 s were used. The specimens were sanded to approximately half thickness of coating. XRD tests were carried out using the Bragg-Brentano method. A flat specimen was purified using acetone and was mounted in the center of the goniometer circle. Based on the X-ray studies, the type of phases present in the newly formed coating and their intensity were determined.

Microhardness profiles were investigated using a ZWICK 3212 B Vickers hardness tester (Zwick, Ulm, Germany) and FM-810 microhardness tester (Future-Tech, Kawasaki, Japan) equipped with FT-Zero automatic indentation measuring software where indentation load was equal to 100 G and loading time was 15 s. Microhardness tests were carried out in direction from the surface to the substrate of specimens both in the axis and border of laser tracks. It is known that the creation of each subsequent laser track affects the previous one. Therefore, checking the microhardness over the entire specimen surface was necessary.

Corrosion resistance were carried out on an ATLAS 1131 EU&IA potentiostat-galvanostat device (Atlas-Sollich, Rębiechowo, Poland). Potentiodynamic method was used and anodic polarization curves were obtained. Based on polarization curves and Tafel slope, the corrosion potential and corrosion current were determined. Tafel slope were plotted using AtlasCorr. Corrosion tests were conducted in a 5% NaCl solution. In this study the reference electrode was a saturated calomel electrode and the auxiliary electrode was made of platinum. The potentiodynamic measurements were performed at a temperature of 22 ℃ with scanning speed of 0.5 mV/s in range from −1.4 V to 0.0 V. The range of the tested potential was selected in such a way that all curves could be analyzed in the range of about ±0.3 V around the corrosion potential (Ecorr). The settling time of specimen in NaCl solution before measurement was 1 h. The corrosion resistance tests were carried out according to PN-EN ISO 17475 norm.

## 3. Results and Discussion

### 3.1. Microstructure, Chemical and Phase Analysis

**Figure 3:**
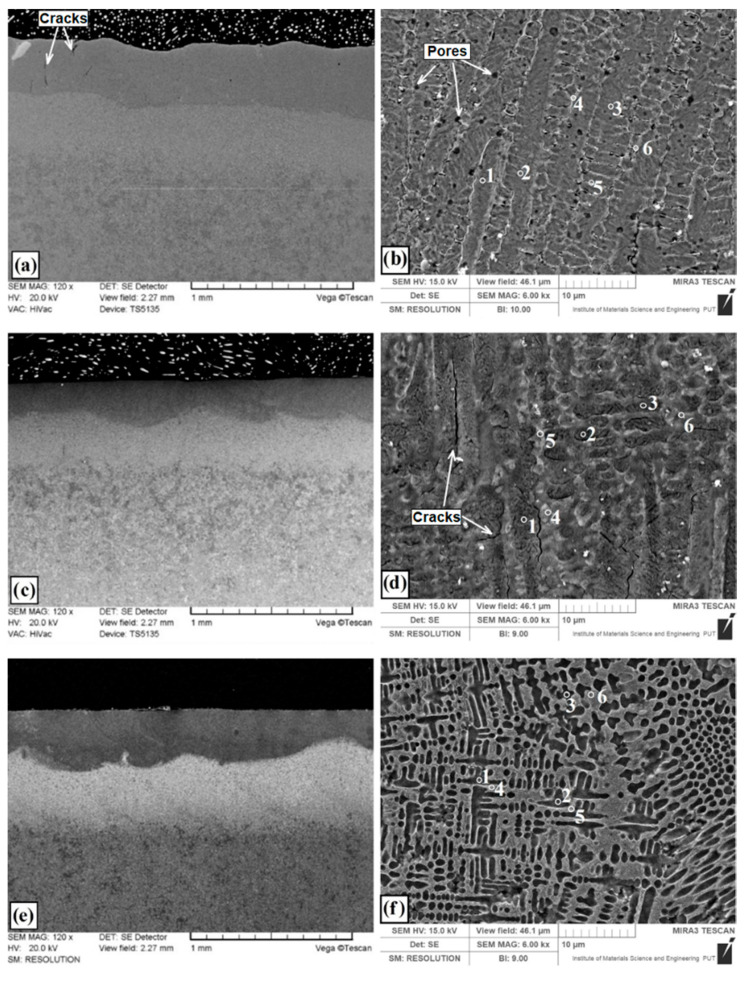
Microstructure of CT90 steel after laser alloying: (**a**) Mo coating; (**b**) Mo coating—magnification remelting zone; (**c**) B coating; (**d**) B coating—magnification remelting zone; (**e**) Mo-B coating; (**f**) Mo-B coating—magnification remelting zone.

Microstructures of coatings after laser alloying are shown in Figure 3. Intensive heating by laser beam and subsequent cooling of the steel and coating containing modifying chemical elements allows to obtain finely crystalline, highly supersaturated solutions. Laser alloying gives possibility to obtain microstructure composed of chemical phases which are difficult to form under normal conditions close to the thermodynamic equilibrium. After laser alloying of pre-coat produced on steel, three zones were observed. First was the remelted zone enriched with modifying chemical element, second was the heat affected zone with martensite and finally third zone of toughened steel substrate. Dimensions of produced laser tracks presented in Table 3 are the result of average from 10 measurements.

It can be seen that in this study the type of pre-coat used had an influence on the laser track depth. Parameters such as heat capacity, thermal conductivity and melting point of pre-coat material are particularly important. The overall microstructure of coating produced using laser alloying of molybdenum pre-coat (Mo-coating) is presented in Figure 3a, while the enlarged area of the remelted zone is shown in Figure 3b. The remelted zone was composed of supersaturated solution of molybdenum in iron where dendrites with long primary axes was visible and their secondary axes in the transverse section had the form of slightly elongated hexagonal cells.

The coating thickness is almost the same throughout the entire specimen area. Under the remelted zone, the heat-affected zone with a martensite microstructure was situated, and even deeper the sorbitic substrate was identified. Single cracks in the remelted zone of the produced coating were visible, which, however, did not cause it to fall off or peel (marked with arrows in Figure 3a). The authors of the paper [20] stated also that the Mo coating was characterized by the presence of a few vertical cracks, mainly from the inside of the coating, which spread towards the surface. In the remelted zone enriched with molybdenum a porosity ranging from 250 nm to 700 nm was identified (marked with arrows in Figure 3b). These porosities are mainly found at the boundaries of solid solution of Mo in Fe. The depth of the remelted zone was from 370 µm to 390 µm. The depth of the entire laser tracks together with the heat affected zone ranged from 840 µm to 855 µm. Notable porosity in the Mo coating was observed in an investigation using heat spraying [14]. It was also found that the use of additional heat treatment reduces pore formations.

Figure 3c presents the microstructure of coating produced by laser alloying of pre-coat containing boron (B-coating). Magnification of the remelted zone is shown in Figure 3d where the presence of borides eutectic with martensite can be found. The arms of the boride eutectic are clearly visible. At high magnifications of microstructure, cracks along the long dendrites can be seen. These cracks were not visible under the optical microscope. The laser tracks have a characteristic parabolic fusion shape, which clearly separates from the heat affected zone (Figure 3c). The depth of the remelted zone of B-coating ranged from 258 μm to 274 μm, while the total tracks depth was from 607–615 μm.

Figure 3e presents the microstructure of coating produced using laser alloying of pre-coat containing both boron and molybdenum (Mo-B-coating). The enlarged area of remelted zone containing a mixture of boron and molybdenum is shown in Figure 3f. The zone includes an iron and molybdenum boride eutectic with martensite. The obtained eutectic was more closely packed and the amount of arms formed during solidification was much larger than in the case of only boron pre-coat. The eutectic formed and the martensite needles were finer, which proves faster heat transfer into the material. The obtained laser tracks have good features of both elements introduced (Mo and B). Boron contributes to the fragmented microstructure and ensures high hardness, while molybdenum due to good thermal conductivity contributes to obtaining deeper laser tracks and hence a thicker coating. The depth of the remelted zone ranged from 335 µm to 355 µm, while the total tracks depth was from 765 µm to 780 µm. Within the microstructure (Figure 3e), the separated areas of the remelted zone and heat affected zone are clearly visible. In heat affected zone of Mo-B-coating smaller grain than in the same area of B-coating was visible. It should be noted that Mo-B-coating microstructure was characterized by lack of cracks. At the same time, no porosity was seen at high magnifications in contrast to Mo-coating.

Several important phenomena occur during the laser alloying process. First, the laser beam hits the pre-coat and induces surface melting. After the pre-coat is completely melted, the substrate partially melts. Then diffusion occurs in melting pool. When the laser beam stops heating the material, the coating solidifies. This process begins from substrate and spreads in the molten pool, forming a coating. In the liquid state, convective movements caused increase of the diffusion in melting pool. This phenomenon results from the existence of three forces: gravity, viscosity and surface tension gradients. In fact, in the melting zone, the temperature is not uniform, and viscosity and surface tension depend on temperature. These gradients induce mass transport (Marangoni effect). The dendrites forming direction in the liquid pool is variable. Convective movements cause the disturbed of solidification process. The dark areas of the melted zone (Figure 3e) indicate the existence of convective movements in this zone. Faulty laser processing conditions causes cracks (B coating—Figure 3d) or porosity (Mo coating—Figure 3b). Enrichment of the surface layer with Mo and B caused decrease of dendritic microstructure size. In this case, microstructure defects were not observed (Figure 3f).

In order to identify the phase composition of the coatings tested, analyses using EDS and XRD methods were carried out. The EDS method is not good enough for determining the amount of boron because it has a relatively weak peak-to-background ratio, and in addition, boron peaks often coincide with carbon peaks. However, there are available papers in which the authors used the EDS method to indicate the boron quantity or its approximate content [22,23]. Some authors obtain a higher boron content in the produced coatings [19], however, it should be noted that in the paper cited the carbon content in the coating was completely omitted.

**Figure 4:**
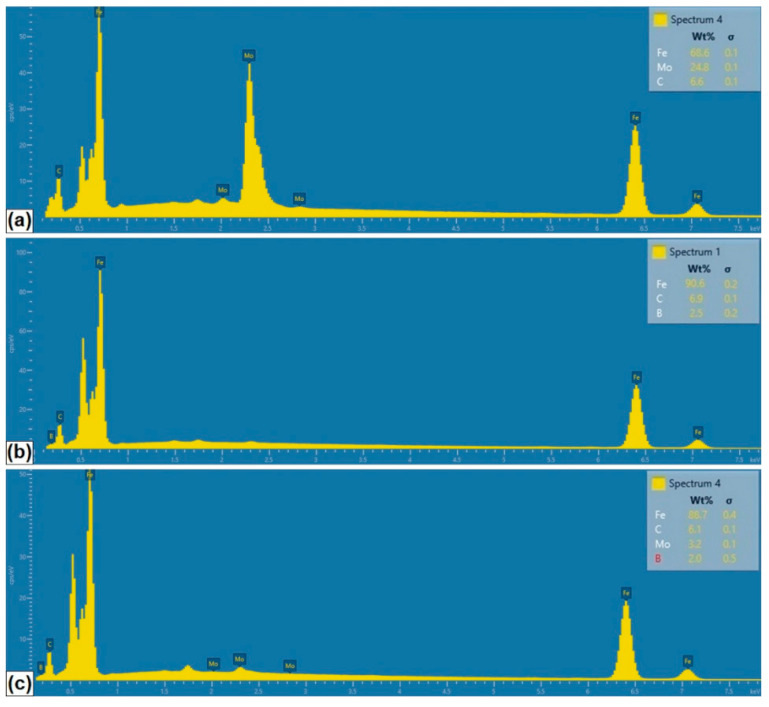
EDS spectrum of produced: (**a**) Mo coating; (**b**) B coating; (**c**) Mo-B coating.

Figure 3b,d,f show the measurement points of significant chemical elements occurring in the produced coatings. The measured values are shown in Table 4, and example spectra for the selected coatings are presented in Figure 4.

**Figure 5:**
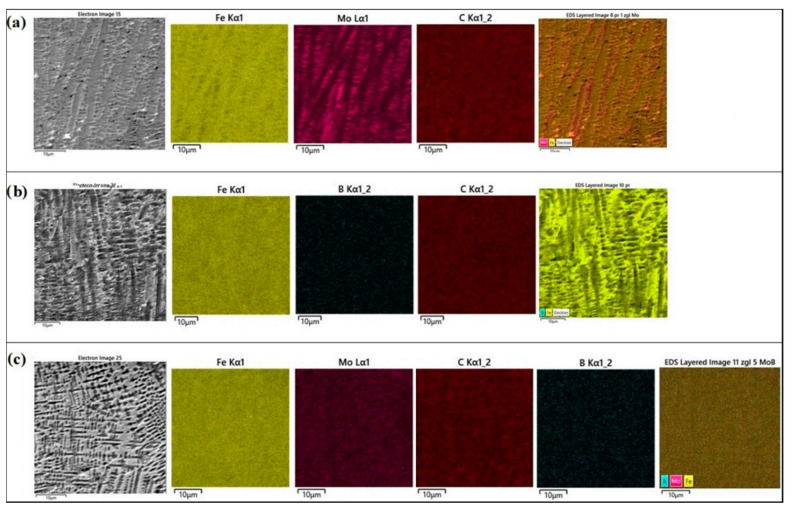
EDS mapping of produced: (**a**) Mo coating, (**b**) B coating, (**c**) Mo-B coating.

In the case of the Mo coating (Figure 3b), the EDS method was limited to three chemical elements that have a significant share in the coating, i.e., iron, molybdenum and carbon. An increased molybdenum content exceeding 10 wt.% in the dendritic skeletal areas was found (Figure 3a), while in interdendritic spaces its content oscillated in the range of 7 wt.%. Also, EDS mapping made for the Mo coating confirmed the increased molybdenum content in the resulting dendritic skeletal microstructure. As can be seen in Figure 5a, an increased molybdenum content can be seen on dendrites, which is also confirmed by EDS point analysis.

**Figure 6:**
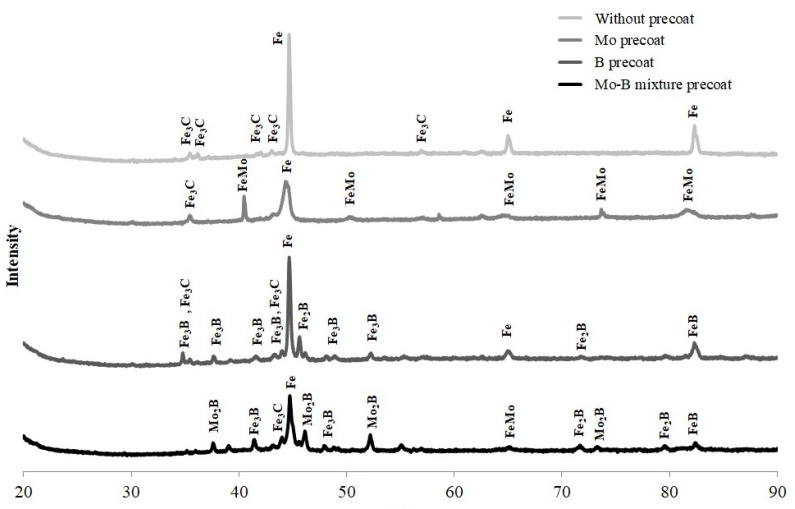
X-ray diffraction spectrum after laser alloying of studied materials.

In Figure 3d the places of point EDS analysis for the boron coating are marked. As in the previous specimens, the measurement points were located in the area of dendrites occurrence and in the interdendritic spaces of the boride-martensitic eutectic. The results are summarized in Table 4, and in Figure 4b an example spectrum is shown. In this case, to assess the approximate share of chemical elements in the coating, iron, carbon and boron were taken into account. Due to the fact that boron is a light element, and its content is distorted by the carbon peaks, these EDS results are only approximate results. It can be assumed that complex phases are formed. It is most likely boron cementite Fe_3_(C, B), where part of the carbon is replaced with boron atoms, which is confirmed by the results of X-ray diffraction analysis (Figure 6). The EDS mapping of the boron coating shows an even distribution of iron, boron and carbon (Figure 5b).

In Figure 3f the places of EDS point analysis for the Mo-B coating in dendritic and interdendritic areas are marked. An increased content of boron and molybdenum was found in the area of dendrites occurrence. In contrast, in interdendritic spaces, the iron amount increases. This happens at the expense of boron and molybdenum, which is confirmed by EDS mapping of this area (Figure 5c). The carbon content for the Mo-B coating presented in Table 3 indicates the overlapping of boron and carbon peaks (Figure 4c). Based on the results obtained, it can be assumed that complex phases Mo_1-x_Fe_x_B are formed, which may correspond to (Fe, Mo)_2_B or Fe_3_(C, B, Mo) phases.

In previous studies [7] where a layer with modifying element was remelted a by laser beam on a steel substrate, the X-ray microanalysis revealed that modifying elements only occurred in the remelted zone, and there was no increase in their content in the heat-affected zone. It was found that the microstructure can be of various shapes, i.e., branched, round or angular [6,7]. As a result of laser processing, the increase of alloying elements from pre-coat (Mo, B) content in remelted zone was observed. It should be noted that the laser processing parameters affect both microstructure and phase composition, and thus the properties of newly formed coatings. The phase composition resulting from laser alloying of pre-coat contained Mo and B was analyzed and the results are shown in Figure 6.

In Mo-coating the presence of FeMo martensite as well as Fe_3_C and Fe peaks were found. In B-coating produced by laser on steel the equilibrium of iron boride phases FeB and Fe_2_B, nonequilibrium of iron boride phase Fe_3_B, and also Fe and Fe_3_C peaks were identified.

In Mo-B-coating the equilibrium phases such as molybdenum boride Mo_2_B and iron borides Fe_2_B and FeB as well as nonequilibrium phases such as iron boride Fe_3_B and also Fe and FeMo peaks were detected, whereas in the CT90 steel after laser alloying the peaks of Fe_3_C and Fe phases were present only. On all three XRD spectra, the peak from Fe was the most intense, which indicates its highest content. In papers [4,11,15,20], the authors confirmed peaks from simple and complex phases corresponding to iron borides and molybdenum borides. These phases were consistent with the B-Mo-Fe ternary equilibrium diagram [24].

### 3.2. Microhardness Profiles

**Figure 7:**
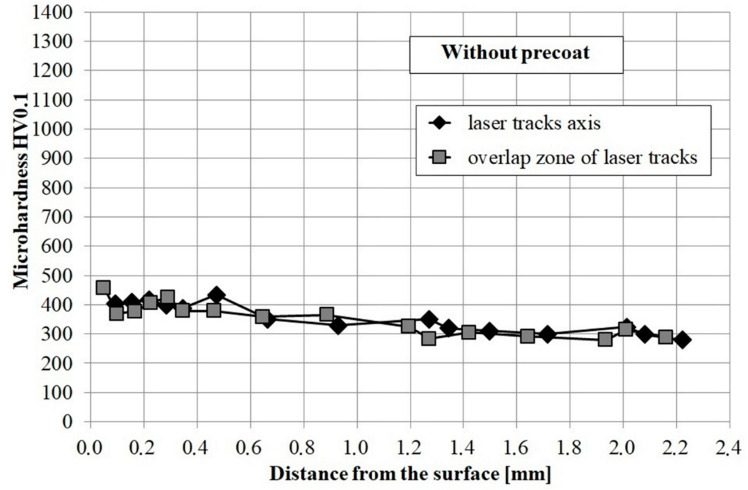
Microhardness of CT90 steel after laser modification.

**Figure 8:**
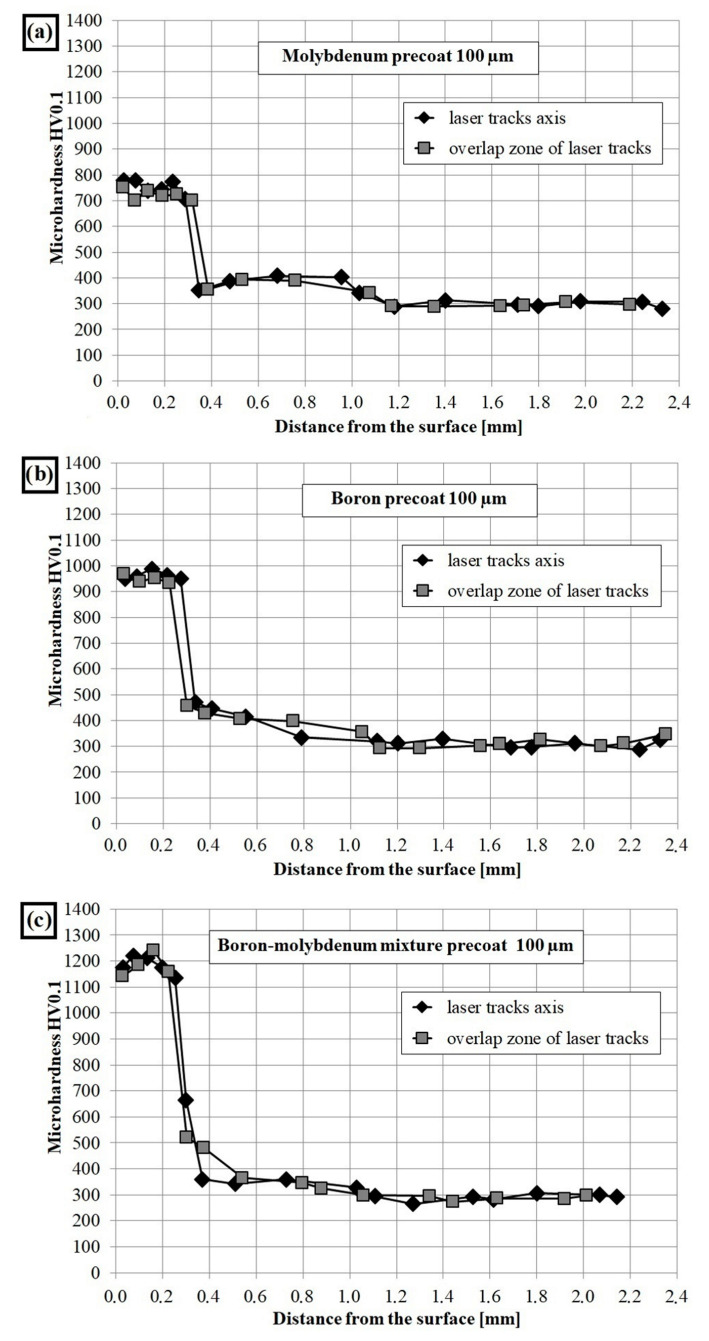
Microhardness of CT90 with laser alloying with pre-coating containing: (**a**) molybdenum; (**b**) boron; (**c**) mixture of boron and molybdenum.

Microhardness profiles of laser modified coating containing molybdenum, boron as well as mixture of boron and molybdenum were compared with the microhardness profiles of CT90 steel after laser remelting using the same laser beam parameters. All the diagrams are presented in Figure 7 and Figure 8. Microhardness was investigated along the axis of the laser tracks and along the overlapping of tracks zone. In both cases microhardness was similar.

The microhardness of CT90 steel after laser modification without any pre-coat was about 400 HV0.1 (Figure 7). The relatively low microhardness could have resulted from the use of high laser beam power. Subsequent laser tracks caused tempering of the previous ones, which resulted in a small increase in microhardness relative to the core.

Figure 8a shows the microhardness profiles of Mo-coating. The tests have shown that microhardness in the remelted zone was about 800 HV0.1 and decreased gradually in the heat- affected zone to a hardness of 400 HV0.1, to finally reach the value of about 300 HV0.1 in the sorbite substrate. The addition of molybdenum caused an increase in microhardness. It can be assumed that during laser processing a solution saturated with molybdenum was formed (which is confirmed by EDS and XRD results). Molybdenum caused increase in hardenability, and thus during laser alloying the coating characterized by higher microhardness was obtained. For comparison, other researchers obtained a molybdenum coating with a hardness from 1100 to 900 HV depending on the laser processing parameters used [20]. While, in paper [13], the hardness on the coating cross-section was in the range from 750 to 650 HV with a laser beam power of 800 W and a scanning speed of 10 mm/s. Figure 8b shows the microhardness profiles of B-coating. As a result of laser alloying the microhardness in the remelted zone was about 1000 HV0.1. In the heat affected zone the microhardness was lower and reaches about 500 HV, while in the core, a value of about 300 HV was found. The boron coatings obtained by laser alloying do not achieve the microhardness such as after diffusion boronizing process but are characterized by greater thickness. The microhardness is affected by the microstructure, which, in turn, changes depending on the parameters of the laser processing. The obtained microhardness values, in the remelted zone, were comparable to the results in other studies [25]. An exception is the case of a coating in which FeB and B_2_O_3_ were produced and its microhardness ranged from 1600 to 900 HV [10]. Each of the base components of the coating is characterized by a different atomic diameter. It can be concluded that the addition of boron (81 pm) or molybdenum (137 pm) to steel substrate consisting mainly of iron (124 pm) have an influence on the change in the crystal lattice and thus, the properties of the coating.

Various microhardness values in remelted zone for Mo-B-coating were probably due to varying thermal capacity of these materials. Average microhardness in the remelted zone of these coatings was approx. 1200 HV0.1 and decreased in the heat affected zone to a hardness ranging from 600 HV0.1 to 450 HV0.1, to finally reach the hardness of steel substrate (Figure 8c).

It should be mentioned that the iron borides and molybdenum borides are characterized by high hardness (Mo_2_B has 1660 HK, and Fe_2_B has 1600 HV). In this study, such results of hardness were not obtained due to the high proportion of iron in the substrate, which is confirmed by the results of EDS and XRD analysis. In other work, B-Si coatings was examined [26]. A lower laser beam power density (33.12 kW/cm^2^) was used. Coatings were made on medium carbon steel. As a result, both boron and B-Si coatings were characterized by smaller thickness. It was found that addition of boron and silicon contribute to formation of hard iron borides phases. The B-Si coating had a higher microhardness than Mo-B coating and reached values up to 1800 HV0.1.

### 3.3. Corrosion Resistance

**Figure 9:**
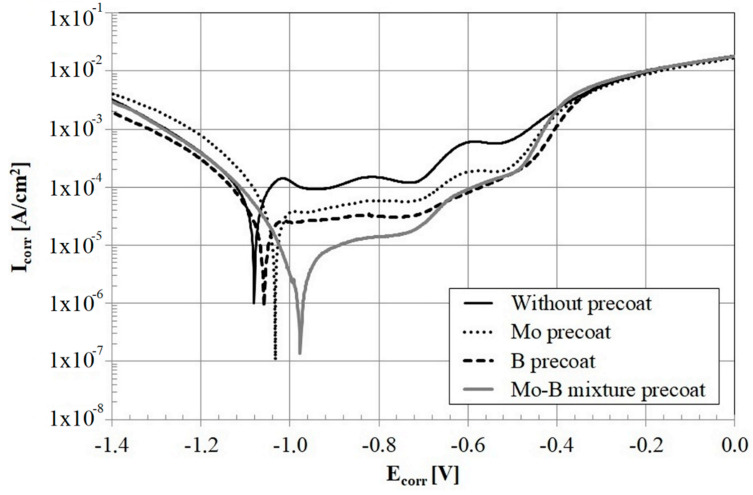
Corrosion resistance of the studied materials and coatings containing molybdenum, boron, and mixture of boron and molybdenum, E_corr_ ranged from −1.40 to 0.0 V.

Results of corrosion tests are shown in Figure 9 where the current density curves are presented as a function of predetermined potential. The values of electrochemical parameters determined based on the analysis of the curves are shown in Table 5. In the tests carried out in a 5% NaCl solution the lowest corrosion resistance was obtained for CT90 steel after laser alloying. Laser modified Mo-coating and B-coating as well as Mo-B-coating showed better corrosion resistance. The best corrosion resistance was observed in the laser modified Mo-B-coating. Despite the occurrence of fine porosities of nanometeric size which were present in the microstructure of the Mo-coating, there is no rapid deterioration of corrosion resistance.

However, the combination of boron and molybdenum significantly improves the resistance of steel covered with such a coating. It can be seen that the corrosion curves are shifted towards lower potentials and lower corrosion current, which is a sign of better corrosion resistance.

The good corrosion resistance is affected by the obtained eutectic microstructure, which is rich in iron borides and molybdenum. The graph in Figure 9 shows a slightly worse corrosion resistance of boron coating than molybdenum coating. It can be assumed that the reduced corrosion resistance was affected by cracks along the long dendrites in the boride-martensitic eutectic structure, visible in Figure 3b. The B-Si coatings [26] showed slightly better corrosion resistance than Mo-B coatings. This can be explained by the fact that described Mo-B coatings were thicker, so the content of iron susceptible to corrosion was higher.

**Figure 10:**
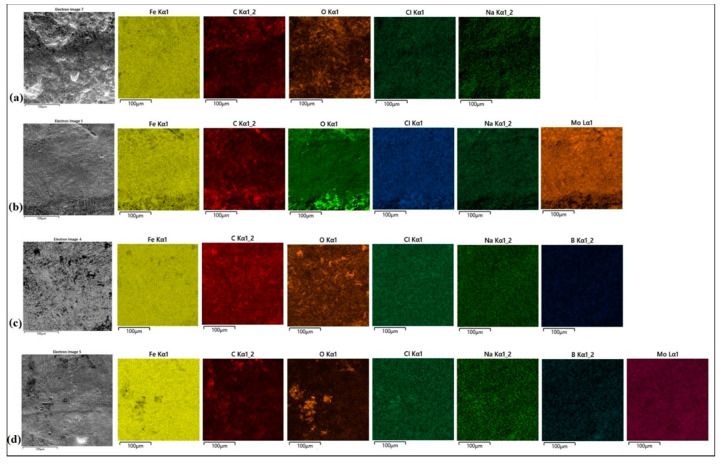
EDS mapping of surface condition after laser alloying and corrosion tests: (**a**) CT90 steel; (**b**) Mo coating; (**c**) B coating; (**d**) Mo-B coating.

Surface conditions after corrosion resistance tests for all type of specimens are shown in Figure 10a–d. The surface of CT90 steel after laser remelting was characterized by a larger number of large-size corrosion pits. Therefore it can be concluded that this surface was more susceptible to corrosion (Figure 10a). There are also corrosion pits on the boron coating, but smaller in size and evenly distributed. Therefore, it can be concluded that the production of boron coating increased corrosion resistance compared to the laser alloying of steel (Figure 10b). No corrosion pitting was found on the Mo coatings (despite numerous porosities) and on the Mo-B coatings (Figure 10c,d, respectively). In addition, to determine corrosion products, all specimens were subjected to EDS analysis.

The EDS mappings are shown in Figure 10a–d. Basic chemical elements from the coating and oxygen responsible for the formation of oxides, as well as chlorine and sodium from the 5% corrosive solution used for the tests were taken into account. Figure 10a shows the surface of a steel specimen after laser remelting. The oxide products appeared on the entire observed area, and the distribution of oxygen was relatively even.

Figure 10b shows the surface of the Mo coating after corrosion tests. Increased oxygen content can be observed mainly in the bottom part of presented area. Fine porosities are visible there. Figure 10c shows the surface condition of boron coating after corrosion testing in NaCl solution. Darker areas are the privileged places of oxide layer growth. In addition, cracks in the microstructure may have contributed to the reduction of corrosion resistance (Figure 9). Figure 10d shows the surface condition of Mo-B coating after corrosion tests. In some areas, an increased oxygen content can be seen, but it does not indicate pitting but only an oxide layer on the specimen surface. No porosity was observed in the microstructure, and the obtained values of potential and corrosion current indicate the best corrosion resistance. In all analyzed specimens subjected to corrosion tests, a multi-phase microstructures were identified, which was confirmed by the XRD results. Based on the obtained microstructure results, as well as EDS and XRD analysis in combination with corrosion tests results, it can be stated that boride-forming chemical elements increase corrosion resistance.

## 4. Conclusions

As a result of a laser alloying process using selected chemical elements and a diode laser beam a newly-formed microstructure consisting of three zones (remelted zone, heat affected zone and substrate) was obtained on a steel substrate. The individual zones were characterized by different properties. Microstructure of the remelted zone obtained by using laser alloying of molybdenum precoat consisted of solid solution of Mo in Fe and was characterized by a small number of cracks and porosity of nanometric size. In the microstructure Fe-Mo, Fe and Fe_3_C phases were detected. The microhardness of this coating was equal to 800 HV0.1 and it showed better corrosion resistance than steel without precoat. The boron coating obtained by using laser alloying of a boron precoat was thinner than the molybdenum coating but was characterized by higher microhardness (approx. 1000 HV0.1). In the remelted zone consisting of boron-martensite eutectic microstructure iron boride phases (FeB, Fe_2_B, Fe_3_B) were detected.

However, it was found that the simultaneous addition of boron and molybdenum into the steel surface has an advantageous influence on the properties of newly-created coatings. The microstructure of the remelted zone consisted of an iron and molybdenum borides eutectic with martensite whose phases have been confirmed by X-ray diffraction. Boron-molybdenum mixture precoating resulted in the formation of a coating of intermediate thickness between molybdenum (the thickest coating) and boron (the thinnest coating).

The combination of these two chemical elements has positive effects on the obtained properties of the coating: a hardness of about 1200 HV0.1 and the best corrosion resistance. The undoubted advantage was also that all prepared coatings had a mild microhardness gradient between remelted zone and substrate, it is influenced by the occurrence of a heat affected zone.

## Figures and Tables

**Table 1:** Chemical composition of CT90 steel used [wt.%].

C	Mn	Si	P	S	Cr	Ni	Cu
0.89	0.30	0.26	0.008	0.009	0.13	0.03	0.04

**Table 2:** Composition of pre-coat pastes.

Paste Composition	B	Mo	B and Mo Mixture
Chemical element [g]	1.0	1.0	1.0/1.0
Sodium water glass [mL]	0.5	0.5	1.0
Distilled water [mL]	1.5	1.5	3.0

**Table 3:** Average dimensions of laser tracks.

Coating	Remelted Zone Depth on Track Axis [µm]	Remelted Zone Depth on Overlapping Zone [µm]	Whole Depth of Laser Track (MZ + HAZ) [µm]
Mo	380	376	850
B	270	215	610
Mo-B	350	292	770

**Table 4:** Results of EDS point analysis of produced: (a) Mo coating, (b) B coating, (c) Mo-B coating.

Coating	Place of Measurement	Fe	Mo	C	B
**Mo**	1	86.1	7.8	6.1	-
	2	86.1	7.7	6.2	-
	3	87.1	6.5	6.4	-
	4	68.6	24.8	6.6	-
	5	73.6	20.0	6.4	-
	6	82.0	11.5	6.5	-
**B**	1	90.6	-	6.9	2.5
	2	91.3	-	7.3	1.4
	3	91.3	-	7.0	1.7
	4	88.5	-	6.7	4.8
	5	90.0	-	6.3	3.7
	6	90.0	-	6.8	3.2
**Mo-B**	1	91.0	1.5	6.5	1.0
	2	90.5	1.7	6.5	1.3
	3	90.5	1.8	6.2	1.5
	4	88.7	3.2	6.1	2.0
	5	87.3	2.4	6.6	3.7
	6	87.6	2.8	6.9	2.7

**Table 5:** Corrosion current and corrosion potential of CT90 steel specimens after laser heat treatment.

Specimen	Current I_corr_ [A·cm^2^]	Potential E_corr_ [V]
**Laser modified CT90 steel**	7.69 × 10^−6^	−1.08 × 10^+0^
**Laser modified Mo pre-coat**	3.51 × 10^−6^	−1.03 × 10^+0^
**Laser modified B pre-coat**	3.21 × 10^−6^	−1.06 × 10^+0^
**Laser modified Mo-B pre-coat**	7.21 × 10^−7^	−9.77 × 10^−1^
